# Post-fever Retinitis With a Positive Weil-Felix Test: A Study From a Tertiary Center in South India

**DOI:** 10.7759/cureus.53162

**Published:** 2024-01-29

**Authors:** Vivek Wani, Arvind Tenagi, Shivanand C Bubanale, Bhagyajyothi B K, Deepashri Mutalik, Chethana Warad

**Affiliations:** 1 Department of Ophthalmology, Jawaharlal Nehru Medical College, Belagavi, IND

**Keywords:** weil-felix test, post-fever retinitis, retinal hemorrrhages, subretinal white lines, ricekttsial infections

## Abstract

Background

Post-fever retinitis (PFR) is reported two to six weeks after fever and affects one or both eyes. Rickettsial fever is one of the most common causes of PFR. This study aimed to report the clinical features and treatment outcomes of PFR cases with a positive Weil-Felix test.

Methodology

In this study, we collected demographic data, fever details, eye symptoms, ocular examination findings, optical coherence tomography (OCT) and fundus fluorescein angiography findings, laboratory findings, and length of follow-up of consecutive PFR cases with a positive Weil-Felix test. All cases were treated with oral doxycycline and prednisolone. Final best-corrected visual acuity (BCVA) and ocular examination findings were recorded. Visual field examination and follow-up OCT results were noted if available.

Results

A total of nine patients (eight males) with a mean age of 39.1 years with fever before ocular symptoms and positive Weil-Felix test were included. Six patients had bilateral disease. The mean initial and final BCVA in the affected eyes was 1.16 and 0.35 logMAR units, respectively (p < 0001). All 15 eyes had typical white retinitis patches and retinal hemorrhages which resolved after treatment. OCT showed hyperreflectivity and inner retinal disorganization over retinitis patches. White subretinal lines were noted in three patients and retinal nerve fiber bundle defect with corresponding visual field defect was seen in one eye.

Conclusions

In this study, PFR due to rickettsia infections has been reported from our region for the first time. Hence, eye specialists in the region should be aware of this entity.

## Introduction

Post-fever retinitis (PFR) is characterized by decreased vision in one or both eyes with white patches of retinitis usually around the optic nerve head and posterior pole with retinal hemorrhages, macular edema, and disc edema [1]. A history of fever about two to six weeks before the onset of ocular symptoms is common [1-3]. PFR cases have been reported after fever due to various causes such as rickettsia, dengue fever, typhoid, malaria, bartonellosis, Lyme disease, West Nile virus, chikungunya, and Zika virus infections [1,4].

Rickettsia as a cause of PFR is suspected when there are typical fundus findings with a history of a tick bite, skin lesions, and fever about two to four weeks before the onset of visual symptoms. However, many patients do not remember tick bites, or, in some cases, they may not notice skin lesions due to dark skin [4]. Rickettsial disease was reported to be the most common cause of PFR in a study from south India, where the diagnosis of rickettsia was based on the results of the Weil-Felix [2].

The definitive laboratory investigations for rickettsia infections include enzyme-linked immunosorbent assay (ELISA), polymerase chain reaction (PCR) to detect rickettsia from body fluids, and immunofluorescence assay [4-6].

However, ELISA, immunofluorescence assay, and PCR tests for rickettsia are not easily available, with the cost being prohibitive in India. Hence, most researchers rely on the Weil-Felix test to make a presumptive diagnosis of rickettsia as a cause of PFR [1,2,4,5]. The usefulness of the Weil-Felix test has been shown in several Indian studies for the diagnosis of systemic rickettsial diseases [7,8]. There are only a few studies on PFR from India, with few describing the clinical and optical coherence tomography (OCT) features of the condition [2,4,9-11]. To our knowledge, there are no reports of PFR due to rickettsia disease from our region. Hence, we are reporting this series of PFR cases with a positive Weil-Felix test from our district in north Karnataka. In addition, we present unique findings of white subretinal lines seen on fundus imaging with OCT findings and PFR causing nerve fiber bundle atrophy with corresponding visual field defect during follow-up.

This study was previously presented as a paper in the annual meeting of the Karnataka Ophthalmological Society on December 9th, 2022.

.

## Materials and methods

This was a prospective, non-randomized, interventional study. The study was conducted in KLES Dr. Prabhakar Kore Hospital, Belagavi, in northern Karnataka, India. The Committee on Human Subject Research of Jawaharlal Nehru Medical College, Belagavi, provided ethical approval for the study (approval number: MDC/DOME/453 dated 24/01/2019).

We examined consecutive cases with decreased vision and a history of fever two to six weeks before the onset of visual symptoms. If patients had typical features of PFR, they were further examined and investigated. We collected the following demographic details for each patient: age, gender, occupation, and address. We collected information about the approximate date of onset of fever, its duration, and the interval between the fever and onset of eye symptoms. In addition, we collected information regarding any systemic diseases such as diabetes, hypertension, and other diseases where relevant.

A thorough ocular examination was performed for each patient at presentation. The ocular examination included best-corrected visual acuity (BCVA) and anterior segment examination by slit-lamp biomicroscopy with particular attention to the presence of cells in the anterior chamber and anterior vitreous. Anterior chamber activity was graded as per the guidelines of Standardization of Uveitis Nomenclature [12]. The intraocular pressure was measured using the Goldmann applanation tonometer in all eyes.

The posterior segment examination was done using slit-lamp biomicroscopy with a 90 D lens and indirect ophthalmoscopy. We noted the following features during the retinal examination: vitreous cells, disc swelling or hyperemia, retinitis patches and retinal hemorrhages, macular edema, hard exudates, subfoveal fluid, and the presence or absence of exudative retinal detachment. In addition, we looked for retinal vascular changes such as sheathing, beading, and arteriovenous changes. We performed OCT in all cases. We used spectral‑domain OCT (3D Maestro OCT, TOPCON, Japan) in our study. We performed fundus fluorescein angiography (FFA) for patients willing to undergo the procedure.

All patients with typical features of PFR underwent blood investigations including the ELISA test for human immunodeficiency virus, *Treponema pallidum* hemagglutination test, Weil-Felix test, peripheral blood smear for malaria parasites, antibody titers for dengue fever, *Toxoplasma *parasite, cytomegalovirus, herpes simplex viruses 1 and 2, varicella-zoster virus, complete blood cell count, and erythrocyte sedimentation rate.

If the Weil-Felix test was positive with antibody titers of at least 1:80, patients were eligible for inclusion in the study. These patients needed to have negative serological tests for other causes of PFR, as mentioned above. Apart from these inclusion criteria, patients needed to have at least three months of follow-up after presentation to be included in the study. The patients also had to provide written consent to be included in the study.

All patients were treated with oral doxycycline 100 mg once per day for three weeks and oral prednisolone 1 mg/kg body weight for seven days. Steroids were then tapered over six weeks. At each follow-up, BCVA and anterior and posterior segment examination findings were recorded. Moreover, OCT was performed during follow-up visits. Visual field testing was done where indicated. The date of the final follow-up and the duration of the follow-up were recorded. Both initial and final BCVA were converted into logMAR units according to the work by Beck et al. [13]. The mean BCVA at presentation and final follow-ups were calculated. The statistical analysis was performed using Student’s t-test to determine if the BCVA at the final follow-up was statistically better than the BCVA at the presentation. The sample size was limited to the availability of cases who presented consecutively with features of PFR with a positive Weil-Felix test.

## Results

In this study, 15 eyes of nine patients (eight males) were included. One patient with clinical presentation resembling PFR but a negative Weil-Felix test was not included in the study. These patients presented from February 2019 to February 2021. Six patients had bilateral affection and three had unilateral affection. The mean age was 39.1 (range = 20- 61) years. All had a fever 7-30 (mean = 23, range = 7-30) days before the onset of ocular symptoms. The mean presenting BCVA in 15 eyes was 1.22 (range = 0.3 to 1.6) logMAR units. The demographic and clinical features are summarized in Table 1

**Table 1:** Demographic and ocular features of patients. AC = anterior chamber; BCVA = best-corrected visual acuity; BE = both eyes; DE = disc edema; HGs = retinal hemorrhages; KP = keratic precipitates; LE = left eye; ME = macular edema; MS = macular star; MFR = multifocal retinitis; RE = right eye; SRF = subretinal fluid; VB = venous beading; VC = vitreous cells

Patient number	Age/sex	Eye involved	BCVA (logMAR) at presentation		BCVA (logMAR) at the final follow-up		Anterior segment	Posterior segment	Disc and vessels	Duration of follow-up (months)
			RE	LE	RE	LE				
1	57/F	BE	1.6	1.6	0.3	0.3	BE: KPs, AC cells	BE: VC, MFR, HGs, ME	No DE	5
2	38/M	RE	1.6	0.0	0.3	0.0	RE: cells	RE: VC, MFR, HGs, ME	No DE	4
3	55/M	BE	1.6	0.8	0.3	0.3	BE: cells	BE: VC, MFR, Hgs, ME LE: MS	BE: No DE RE: VB	6
4	61/M	BE	0.3	1.5	0.0	0.3	BE: cells RE: KPs	RE: VC LE: MFR, HGs, VB, ME	RE: DE LE: VB	4
5	24/M	BE	0.8	1.5	0.0	0.5	BE: cells	BE: VC, MFR, HGs, SRF, MS	BE: No DE LE: VB	9
6	24/M	BE	1.0	1.6	0.3	0.5	RE: No cells LE: cells	BE: VC, MFR, HGs, MS, ME LE: SRF	BE: DE, VB	4
7	32/M	BE	1.6	1.4	0.5	0.2	BE- no cells	BE: VC, MFR, HGs, ME	BE: DE	6
8	41/M	LE	0.0	1.0	0.0	0.3	LE no cells	LE: VC, MFR, HGs, SRF	LE: DE	12
9	20/M	LE	0.0	0.3	0.0	0.0	LE cells	LE: VC, MFR, HGs, ME	LE: No DE	24

**Figure 1:**
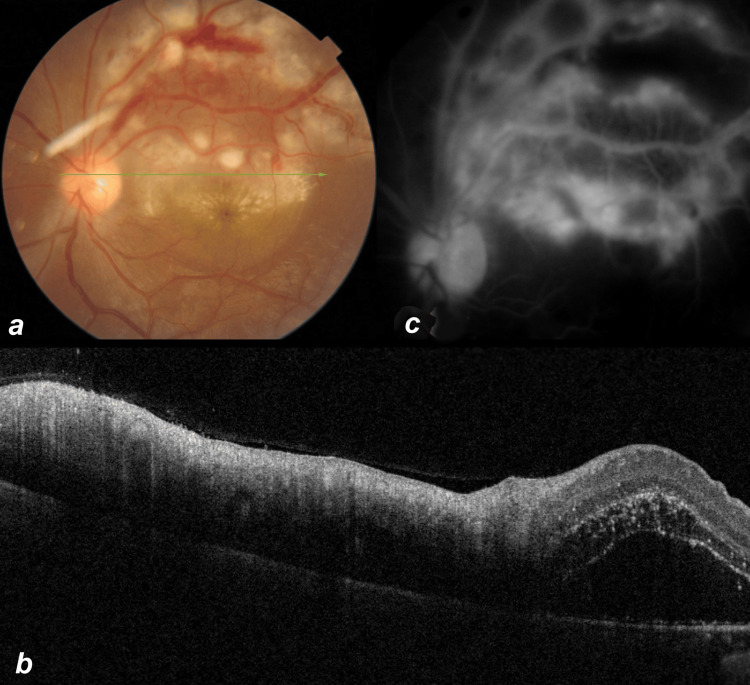
Color fundus, optical coherence tomography, and fundus fluorescein angiography features at presentation. Color photo of the left eye of patient 5 at presentation with multifocal retinitis, retinal hemorrhages, macular star, and venous beading (a). Optical coherence tomography shows hyperreflectivity in the superficial layers of the retina over retinitis patches and subfoveal fluid(b). Late-phase fundus fluorescein angiography shows hyperfluorescence over retinitis patches with vessel wall staining in the late phase (c).

A total of 13 eyes had anterior chamber cells (usually +1 or +2), with three eyes having small keratic precipitates. Anterior vitreous showed cells in all 15 eyes. All 15 eyes had typical multifocal white patches of retinitis and retinal hemorrhages of varying degrees (Figure 1, Panel a).

In almost all cases, the patches were mainly around the disc and along the temporal arcades. In a few cases, white retinitis patches were also seen in the mid-periphery. The disc was edematous in some if the white patches were adjacent to the disc. Macular edema was seen clinically in 14 eyes, and four of these cases had a macular star. Veins were dilated and showed beading (Figure 1, Panel a) in the affected part in five eyes. Serous retinal detachment over the macular area was seen in four cases which was confirmed by OCT (Figure 1, Panel b). OCT showed hyperreflectivity and inner retinal disorganization over the white patches (Figure 1, Panel b). FFA was done in a few cases and showed early hypofluorescence over the white lesions. The later phases of FFA showed leakage and staining of the lesions (Figure 1, Panel c).

One patient was a student of agricultural sciences and had gone for insect collection in the fields, as part of an entomology posting. He gave a history of a skin lesion over the leg after his field visit. None of the other patients gave a history of maculopapular rash or other skin lesions. Another patient was an engineer working in solar power fields. The other seven patients were farmers. The laboratory investigations were negative in all nine patients for dengue fever, *Toxoplasma *parasite, cytomegalovirus, herpes simplex viruses 1 and 2, varicella-zoster virus, *Treponema palladium* hemagglutination, dengue fever, and human immunodeficiency virus. The Weil-Felix test was positive in all nine patients. Two patients were positive for antigen OX 2, two for OX K (scrub typhus), three for both OX 2 and OX K, one for OX 2 and OX 19 (Indian tick typhus), and one for all three antigens. The interpretation of the Weil-Felix test was done as per Mahendradas et al. [1].

**Figure 2:**
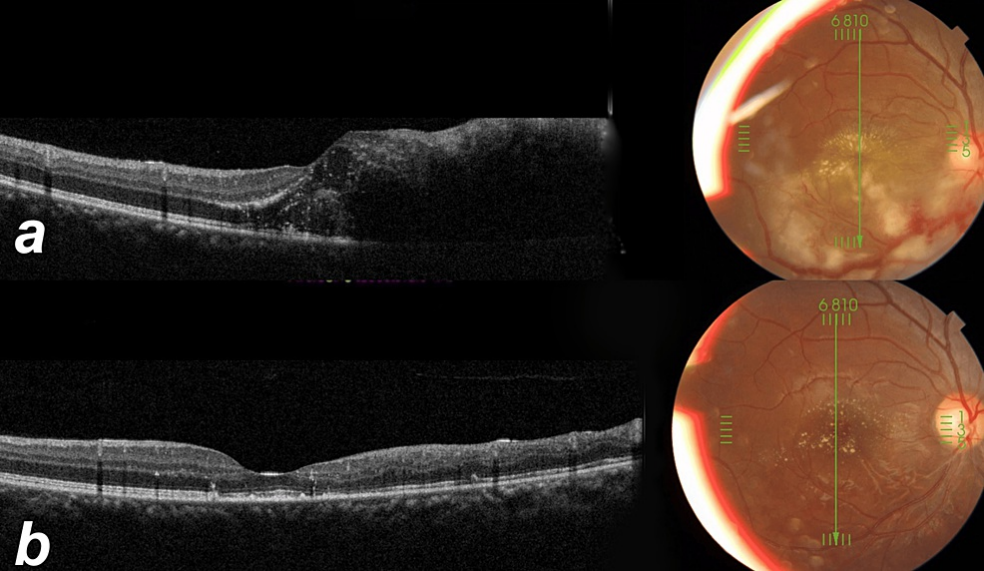
Color and optical coherence tomography at the presentation and follow-up at three months. The right eye of patient 6 (a) at presentation and (b) after three months of presentation.

Follow-up OCT and fundus examination showed resolution of hyperreflective patches of retinitis, hemorrhages, and subretinal fluid in all cases. Figure 2 shows a representative case at the presentation and three months after the presentation during follow-up.

**Figure 3:**
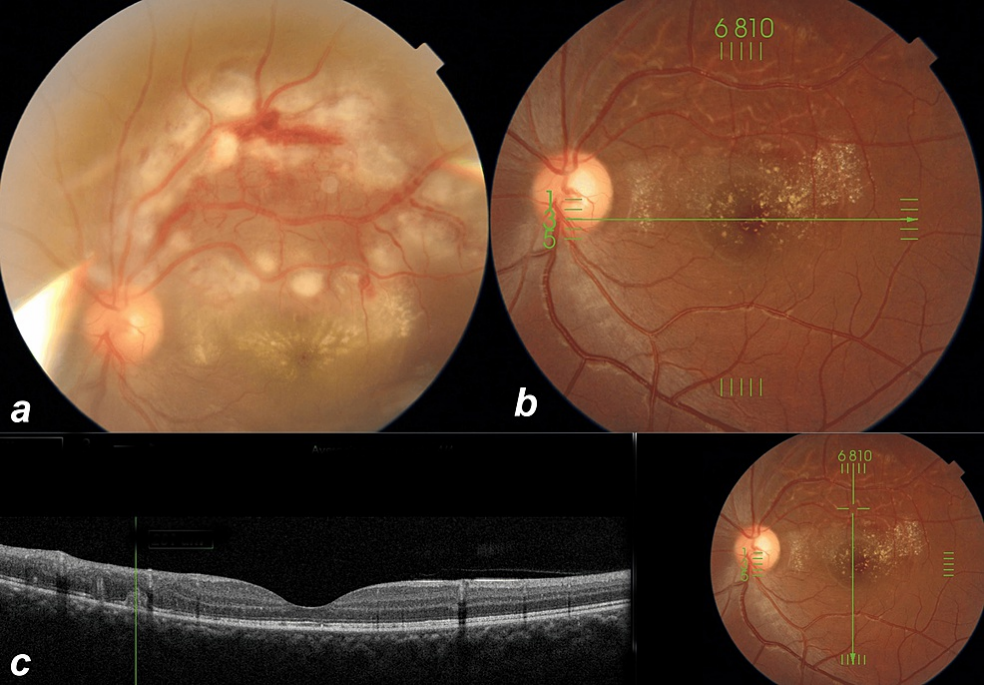
White subretinal lines with corresponding optical coherence tomography. The left eye of patient 9 at presentation (a) and three months later with white subretinal lines (b) and optical coherence tomography over the white subretinal lines (c).

Three patients (5, 6, and 9) showed multiple white subretinal lines in the area of retinitis on fundus examination during follow-ups (Figure 2c, Figures 3a, 3b). The OCT showed the white lines to correspond to hyperreflective material in the subretinal space (Figure 3c).

**Figure 4:**
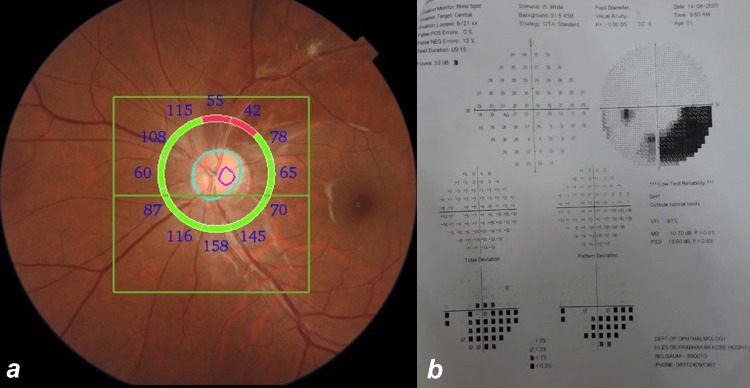
Retinal nerve fiber atrophy and the corresponding visual field defect. The left eye of patient 9 showing (a) retinal nerve fiber layer thinning and (b) the corresponding visual field defect.

In patient 9, we also observed retinal nerve fiber atrophy in the area of previous retinitis, with OCT showing retinal nerve fiber thinning (Figure 4a). Visual field examination showed a corresponding field defect (Figure 4b) one year after the presentation.

Follow-up ranged from three to 24 (mean = 8.2) months. The initial mean BCVA of 1.22 (range = 0.3 to 1.6) logMAR units improved to 0.29 (range = 0.8 to 0.00) logMAR units (p < 0001 compared to presenting BCVA) at the last follow-up. The mean Snellen equivalent of initial and final BCVA in logMAR units were 6/100 and 6/12, respectively.

## Discussion

To our knowledge, there are no reports of PFR from north Karnataka, although rickettsia as a cause of fever has been reported [8]. Although several causes of PFR have been reported in the literature, no study has reported the presence of causative organisms either in the retina or vitreous [1]. Rickettsial fever as a cause of PFR has been reported in India [2,4,9-11].

The average age and preponderance of males are comparable to other studies from India [2,4,10]. The occupation of the patients has not been investigated in many studies; all our patients except one were farmers or worked in the field. These cases point to the possible exposure to tick bites and rickettsial infection. A seasonal occurrence of PFR due to rickettsia has been reported [2,10]. Only one out of nine patients gave a history of skin lesions or rashes, which was similar to another study [10].

The diagnosis of PFR due to rickettsia in our cases was based on typical clinical features, a history of fever two to four weeks before the onset of visual symptoms, and a positive Weil-Felix test. The Weil-Felix test is not specific to rickettsial organisms but is affordable in most centers. The interpretation of the Weil-Felix test was done as per Mahendradas et al [1]. The ELISA test and immunofluorescence test for rickettsial disease are confirmatory but are not easily available and are costly. Hence, clinicians rely on the Weil-Felix test to diagnose rickettsial retinitis as a cause of PFR [4,5].

The presenting visual acuity was poor in most affected eyes in our series. The mean Snellen BCVA in 15 eyes was 20/330 or 1.22 logMAR units. The vision was affected due to macular edema, subretinal fluid, retinitis patches near the macula, disc edema, or neuroretinitis. Kawali et al. in a case series of 10 patients, reported much better Snellen BCVA at the presentation of 20/60 [4]. The anterior uveitis in the form of cells in the anterior chamber was seen in 11 of the 15 eyes. The grading of anterior chamber cells was always 1 or 2, as noted in other studies as well [4,10,11]. Small keratic precipitates were also seen in three eyes. Cells were seen in the anterior vitreous in all eyes on slit-lamp biomicroscopic examination. Hence, clinicians should recognize that PFR retinitis is a form of panuveitis.

Fundus features were characterized by the presence of usually multifocal cotton wool-like patches of retinitis mainly around the disc and along the temporal arcades and posterior pole. There were retinal hemorrhages mainly of the superficial type usually located near the white retinitis patches. Disc edema, macular edema, subretinal fluid, macular star, venous dilatation, and beading of veins were other common findings in our cases.

Scheie reported ocular findings in 451 combatants of World War II who contracted scrub typhus in the Indian subcontinent [14]. The reported retinal findings were venous dilatation, a sausage-like appearance of veins, disc edema, hemorrhages, and cotton wool-like spots [14]. Surprisingly, the presence of retinitis patches described as fluffy cotton wool spots was noted only in 4.9% of 451 patients. However, as the study was mostly a review of records with no fundus photographs available during those times, these important lesions of white retinitis patches might have been underreported. In our series, the sausage-like appearance of veins or venous beading was noted in five eyes. However, venous beading has not been mentioned in any of the studies referred to above [2,4,5,10].

We did not see vascular sheathing or vasculitis in any of the 15 affected eyes. Kawali et al. also did not report any eye with vascular sheathing [4]. However, Chawla et al., in a case report, noted perivascular exudation in both eyes of a patient [11].

The OCT features were mainly in the inner retina. OCT showed hyperreflective inner layers with disorganization of inner layers. The inner layer hyperreflectivity also resulted in after shadowing. The OCT helped in confirming the presence of subretinal fluid over the macular area in five cases in our series. These features on OCT have also been reported by others [2,4,10]. The findings of FFA in our cases were similar to those reported in other studies.

There are some reports from other regions of the world where rickettsial disease has been reported as a cause of retinitis [15,16]. Khairalla et al. reported posterior segment manifestations in 30 cases of Mediterranean spotted fever caused by *Rickettsia conorii* [15]. They observed that the majority of patients were asymptomatic and those with visual disturbances presented two to five days after suffering from fever [15]. This is in contrast to our study in which all patients with presumed rickettsia retinitis were symptomatic in at least one eye. Balasundaram et al. reported 12 cases of Indian tick typhus caused by *Rickettsia conorii*, all of whom presented with decreased vision in at least one eye [10]. The interval between an episode of fever and the onset of visual complaints was much longer in our study at two to four weeks in eight cases and seven days in one case. Other studies from India observed that the interval between fever and the onset of visual symptoms was usually between two to four weeks [4,10].

Both Khairallah et al. and Kahloun et al. observed the presence of white retinitis patches in 75% of the eyes [15,16]. Other retinal findings were similar to the present study. However, both studies reported branch retinal artery occlusion in one eye each which was not observed in our study. However, Kawali et al. observed branch retinal artery occlusion in one case [4]. This indicates that, though retinal veins are mainly affected with dilatation and beading, the retinal arteries can also be affected in PFR due to rickettsial disease.

In this study, we observed the unique finding of white subretinal lines in some cases during the recovery of retinitis. These findings have been reported in only one study from India which they described as outer retinal folds [17]. However, in our series, these lines were subretinal, had a slightly curved shape, and were about two to three disc diameters in length. Using OCT, they corresponded to hyperreflective material that was located in the subretinal space placed internal to the retinal pigment epithelium line. This may be inflammatory material probably arranged in lines due to their deposition along the underlying choroidal blood vessels. They disappeared over time, as shown in Figure 4.

In patient 9, we noticed a retinal nerve fiber bundle defect along the superior pole of the disc and over the superotemporal arcade where the PFR lesions were present (Figure 1, Figure 4a). Initially, the visual field examination did not detect the visual field defect, but six months later, there was a definite sickle-shaped inferior visual defect that corresponded with the nerve fiber bundle defect (Figure 4b). A study reported retinal nerve fiber bundle defect and focal retinal thinning on OCT but did not report a corresponding visual field defect [18].

The visual recovery in most patients was good in our series. Visual recovery occurs over three to five months with a resolution of retinal lesions. Most studies and case reports reported good visual recovery in patients with PFR due to rickettsial disease [2,4,10,11,18,19].

Our study has the shortcoming of presuming the diagnosis of rickettsiosis based on the positive Weil-Felix test which is not a definitive test. Unfortunately, we were not able to perform immunological tests to confirm the diagnosis of rickettsial disease as the tests are difficult to access and costly. Many studies from India have relied only on the Weil-Felix test to diagnose rickettsial disease as a cause of PFR for the same reasons [4,2,9]. We ruled out other common causes of PFR by performing respective diagnostic tests. The strength of the study is the reporting of a series of cases with PFR with presumed rickettsiosis from a hitherto unreported region. The number of patients in the series is comparable to other studies from the region [4,10].

Regarding the treatment of PFR due to rickettsia, we treated with oral doxycycline and steroids with good results. Whether the addition of steroids to doxycycline treatment is beneficial is controversial, but most clinicians start oral steroid therapy along with doxycycline therapy [1,2,4,15,16]. The cause of retinitis in rickettsia remains unclear as to whether it is a direct infection of retinal tissues or an inflammatory reaction to the rickettsial proteins. Given its occurrence after the episode of fever and good response to steroids, many studies view this is an immune response to systemic infection [4,15].

## Conclusions

It is important to recognize the probability of rickettsial etiology in all cases of PFR and order appropriate tests. Although it is desirable to conduct specific ELISA tests to confirm rickettsial infection, the Weil-Felix test was used in our series due to financial constraints. As PFR due to rickettsia is mostly self-limiting and has a good visual prognosis, it is important to diagnose it to avoid unnecessary investigations and treatment. Unique findings such as white subretinal lines in the area of retinitis and retinal nerve fiber bundle defects may be observed during follow-up. We have shown that, in our part of Karnataka, rickettsial disease can cause PFR. This article highlights this issue to make the eye specialists of the region aware of this entity.
